# Discovery that cells have plasma membrane portals called porosomes that govern secretion

**DOI:** 10.15190/d.2023.15

**Published:** 2023-10-22

**Authors:** Elisa A. Liehn

**Affiliations:** Professor of Translational Cardiology, University of Southern Denmark, Odense, Denmark

**Keywords:** Porosome, membrane portal, discovery.

## Abstract

A large number of products are synthesized and packaged in membrane vesicles to be secreted from cells to carry out essential physiological functions such as nerve transmission, digestion and immune response. How do cells secrete with great precision a portion of the vesicle contents?These questions have been answered through the work of Dr. Bhanu P. Jena, a cell physiologist and chemist at Wayne State University School of Medicine in Detroit Already in the mid 1990s he discovered that pancreatic acinar cells possess secretory portals (porosomes) at the cell plasma membrane that govern the transport and secretion of digestive enzymes. During the next twenty-five years, Jena characterized in great detail the molecular mechanisms underlying this secretory process. He also showed that similar “secretory portals”, or “porosomes”, are present in all cell types including endocrine cells secreting hormones and brain neurons secreting neurotransmitters.The principles discovered and described by Bhanu P. Jena turned out to be universal, operating similarly in all animal cells. A number of human hereditary diseases are caused by mutations in some of the nearly 30 proteins composing the porosome complex. Jena’s discovery of the porosome, in addition to providing a deep understanding of cell secretion, has also contributed to the establishment of a drug development platform (https://www.porosome.com) for the treatment of a wide range of diseases. Among the therapeutic application is porosome reconstitution in stem cell derived beta cells, for the treatment of Type 1 diabetes and holds great promise for the cure of cystic fibrosis.

## Cellular nanomachines like the “porosome” serves important life functions

An adult human being is made up of approximately 38 trillion cells that communicate with each other via secretion to establish homeostasis and sustain life. The cell is analogous to a well-organized city, with thousands of reactions and millions of interactions occurring at any given moment. The cell nucleus, for instance, contains nearly 6 feet of the genetic material DNA to governs all functions of the cell. Hence each human has enough DNA to span more than 60 times the 93 million miles between the earth and the sun. This provides a glimpse of the enormity of inter-cellular information transfer via secretion, required for maintaining homeostasis. Transcription and translation result in the production of over a billion protein molecules in each cell, some of them giving rise to the various cellular nanomachines, -the cellular work horses that sustain life. These nanomachines measure just 15–150 nm; hence, 10,000 of 15 nanometer-sized nanomachines could fit in the thickness of a human hair.

Among the key cellular nanomachines discovered are the “porosome” or “secretory nanomachine” at the cell plasma membrane for the energy-dependent fractional release of intra-vesicular contents from cells; “chaperonin” the energy-dependent protein-folding machinery in cells; “proteasome,” the energy-dependent garbage disposal in cells; “ribosome,” the protein synthetic machinery in cells; “ATP synthase,” the nanomachine that generates cellular energy; and “myosin,” the cellular molecular motor for movement and transport.

## How do cells expel a fraction of secretory vesicle contents during secretion? 

**Figure 1:**
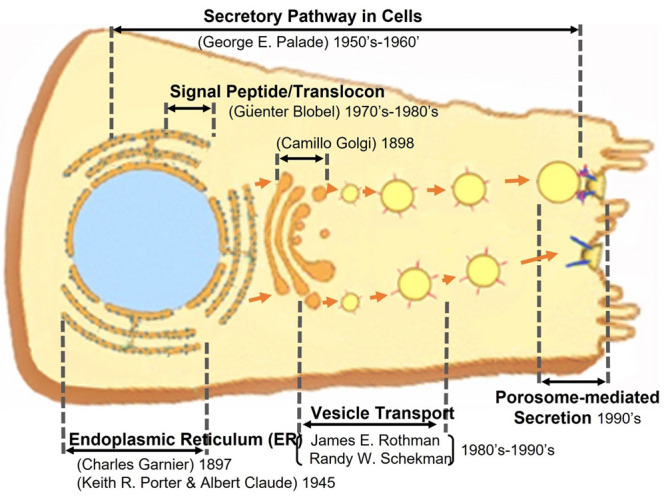
The secretory pathway in cells. Schematic drawing of the key discoveries made on the various cellular structures involved in cell secretion.

It was for a long time a puzzle how a fraction of the secretory vesicle content is released (as observed in electron micrographs of cells following secretion), if according to popular belief, secretory vesicles were to completely merge at the cell plasma membrane, enabling release of the entire vesicle content from the cell during secretion. Bhanu P. Jena was going to solve this puzzle. In 1990 he joined the famous cell biology laboratory of James D. Jamieson, a former student of George E. Palade at Yale University School of Medicine in New Haven. Here, and prior to that at the Rockefeller University in New York, for decades, scientists had studied the structure of the cell and the principles for the transport of newly synthesized proteins out of the cell. This work earned first George E. Palade the Nobel Prize in Physiology or Medicine in 1974 (which he shared with the Belgian scientists Albert Claude and Christian de Duve), and the unshared 1999 Nobel Prize in Physiology or Medicine to one of Palade’s student Günter Blobel. Bhanu P. Jena remained at Yale until 2000, first as a Fellow then as an Assistant Professor investigating cell secretion, and frequently discussing the puzzle of fractional vesicle release during secretion with George E. Palade and Günter Blobel^1,2^ (Figure 1).

## The “porosome” hypothesis

**Figure 2:**
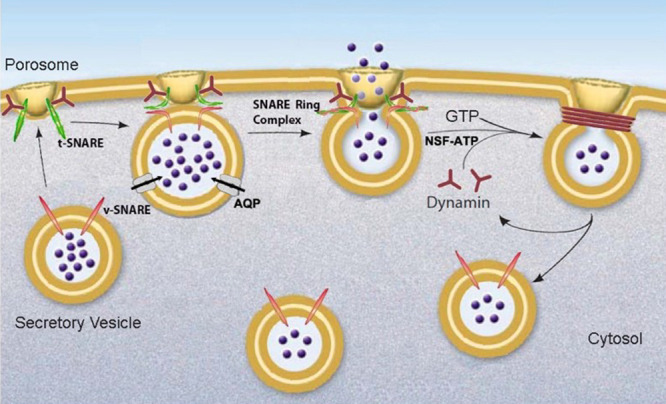
The “porosome-mediated secretion hypothesis”. Schematic drawing of porosome-mediated fractional release of intra-vesicular contents from the cell during secretion. Secretory vesicles dock at the porosome base via t-SNAREs present at the pososome base and v-SNAREs present at the secretory vesicle membrane to form a t-/v-SNARE ring complex, establishing continuity between the opposing membranes (fusion pore) through which pressurized intra-vesicular contents [intra-vesicular pressure established via active transport of water through aquaporin or water channels (AQP) at the secretory vesicle membrane] are expelled to the outside during cell secretion. Following secretion, the t-/v-SNARE rosette complex is disassembled by NSF-ATP and the fused lipid membrane is cleaved by dynamin-GTP. The resultant partially empty vesicle then dissociates from the porosome at the cell plasma membrane (Courtesy of Prof. Bhanu P. Jena).

To understand how a secretory vesicle could transiently dock and fuse at the cell plasma membrane without compromising integrity of both the plasma membrane and secretory vesicle membrane, and how intra-vesicular content is fractionally expelled, was a major focus of the Jena group. Jena opined that there needs to be a stable structure at the cell plasma membrane that would facilitate precision docking of the secretory vesicle, enable fusion to establish continuity between the opposing membranes (fusion pore) without complete vesicle merger, expel the required amounts of intra-vesicular contents, and disengage (endocytose) from the cell plasma membrane (Figure 2).

Bhanu P. Jena’s research was built on the traditions of Palade, Jamieson and Blobel´s laboratories. In particular, Jena studied the nanoscale dynamics of the plasma membrane and the underlying secretory vesicles in live cells during secretion, using Atomic Force Microscopy (AFM). Jena’s observation using AFM, of 100-150 nm pores at the apical plasma membrane in live pancreatic acinar cells, and pore dilation following stimulation of cell secretion, suggested the presence of secretory portals. The return of these pores to their resting size following completion of secretion, further demonstrated them to be permanent structures at the cell plasma membrane. Exposure of cells to the actin depolymerizing Cytochalasin, results in collapse of the pore opening and a loss in cell secretion^3^. Similarly, the observation using AFM, the swelling of secretory vesicles docked at the cell plasma membrane in live cells on stimulation of cell secretion, followed by secretion and the consequent decrease in vesicle size, further confirmed that cells partially discharge intra-vesicular contents during secretion, retaining vesicle identity. Based on these elegant experiments, Jena hypothesized in 1996, the presence of secretory portals or “porosomes” at the cell plasma membrane where secretory vesicles transiently dock, fuse, and release in a precise manner, a measured amount of intra-vesicle content, dictated by the extent of vesicle swelling (Figure 2). During the next twenty years, Jena and coworkers, step by step characterized the molecular details of the porosome-mediated cell secretion processes. Eventually it was shown that the “porosome” hypothesis was both correct and universal, since the processes operate in the same way in all cells.

## “Porosome-mediated” cell secretion

In collaboration with other research groups, Bhanu P.Jena was soon able to show that similar porosome-mediated secretion occurs in other cellssuch as the mast cell^4^ and the growth hormone cellin the pituitary gland^5^. On the basis of these results, Bhanu P. Jena formulated in 2002 the general principles for porosome-mediated cell secretion. Each cell type carries in its porosome structure theunique information needed to specify its properlocation and the targeting of specific secretory vesicles to dock and transiently fuse to release intra-vesicular contents during cell secretion. This was a major breakthrough and a paradigm shift in our understanding of the secretory process in cells. In recognition, Bhanu P. Jena received honorary doctorate in Medicine from Babes Bolyai University in Cluj Napoca, Romania, jointly with George E. Palade and Günter Blobel, in 2003. Since its discovery, the porosome has been intensely investigated by Jena^6-12^ and other laboratories. Porosomes have been found to be present in every cell examined, from cells of the exocrine pancreas secreting digestive enzymes, to hormone secreting endocrine cells and in neurons for neurotransmitter release. Porosomes are cup-shaped lipoprotein structures ranging in size from 150 nm in the exocrine pancreas and endocrine cells, to 15 nm at the nerve terminal. Porosomes are composed of nearly 30 proteins, they have been isolated from a number of cell types and functionally reconstituted both into lipid membrane and live cells^6-12^ .

In the past two decades, the Jena laboratory has additionally contributed to our understanding of the fundamental molecular process involved in Ca+2 and SNARE-mediated membrane fusion^13-16^, and on secretory vesicle volume regulation required for fractional release of intravesicular contents^17-21^ via the porosome during secretion. This process enables cells to precisely regulate the discharge of a portion of their intra-vesicular contents during a secretory episode, while retaining integrity of both the vesicle membrane and the cell plasma membrane. Studies from many laboratories demonstrate altered porosome proteins to result in a wide range of secretory defects and diseases.

## Significance of Jena’s discovery

Bhanu P. Jena’s discovery has had an immense impact on modern cell physiological research. Secretion is a highly regulated fundamental cellular process in living organisms, from yeast to cells in humans. Cellular cargo such as neurotransmitters in neurons, insulin in beta cells of the endocrine pancreas, or digestive enzymes in the exocrine pancreas, are all packaged and stored in membrane-bound secretory vesicles that dock and fuse at the cell plasma membrane to release their contents during secretion. The prevailing view for over 70-years was that secretory vesicles completely merge with the cell plasma membrane, emptying the entire vesicular contents outside the cell during secretion. The attention given to this all-or-none complete vesicle merger mechanism of cell secretion, held back progress in the field, even though there had been accumulating evidence favoring fractional release. For decades, the generation of partially empty secretory vesicles had been observed in electron micrographs in cells following a secretory episode, suggesting fractional release of intra-vesicular contents during cell secretion. How fractional release of intra-vesicular contents during cell secretion is accomplished with great precision, was finally elucidated by the pioneering studies by Bhanu P. Jena. This was a major breakthrough and a paradigm shift in our understanding of the secretory process in cells.

## Future applications

The discovery of the ‘porosome’, the cell's secretory apparatus, has provided a platform^22^ [https://www.porosome.com] for entry into a new era in drug development, design, and therapy. Many diseases, such as Cystic Fibrosis, Type I and Type II Diabetes, neurological disorders, immune disorders and various cancers, result from secretory defects in the porosome. These diseases can now be addressed by incorporating the native functional porosome machinery into diseased cells, or by using small molecules and nanobodies directed at porosome proteins, to modulate the cell's secretory activity and treat the disease. Functional reconstitution of the porosome complex which has been achieved both in a lipid membrane and in live cells, therefore offering significant promise for therapeutic interventions and high-throughput drug screening. Similarly, tissue-specific porosome-targeted therapy has the potential to revolutionize medicine development by enabling the creation of an unprecedented variety of medicines tailored to a wide spectrum of secretory disorders that result in different diseases. Development of tissue-specific medicines targeted to porosomes not only advances therapeutic precision but also significantly elevates drug safety. Targeted toward the porosome, these medicines aim to effectively mitigate toxicity, minimize associated side effects, and mitigate various risks across the entirety spectrum of drugs in treating secretory disorders.
